# Nanoscratch-induced formation of metallic micro/nanostructures with resin masks

**DOI:** 10.1186/s11671-023-03857-x

**Published:** 2023-05-27

**Authors:** Mingyong Xin, Qihui Feng, Changbao Xu, Licong Cui, Jie Zhu, Yinkai Gan, Bingjun Yu

**Affiliations:** 1 Electric Power Research Institute, Guizhou Power Grid Co., Ltd., Guiyang, 550002 Guizhou China; 2 School of Mechanical Engineering, Southwest Jiaotong University, Chengdu, 610031 Sichuan China

**Keywords:** Nanoscratch-induced directional deposition, Metallic micro/nanostructures, Keto-aldehyde resin mask, Silicon

## Abstract

Metallic micro/nanostructures present a wide range of applications due to the small size and superior performances. In order to obtain high-performance devices, it is of great importance to develop new methods for preparing metallic micro/nanostructures with high quality, low cost, and precise position. It is found that metallic micro/nanostructures can be obtained by scratch-induced directional deposition of metals on silicon surface, where the mask plays a key role in the process. This study is focused on the preparation of keto-aldehyde resin masks and their effects on the formation of scratch-induced gold (Au) micro/nanostructures. It is also found that the keto-aldehyde resin with a certain thickness can act as a satisfactory mask for high-quality Au deposition, and the scratches produced under lower normal load and less scratching cycles are more conducive to the formation of compact Au structures. According to the proposed method, two-dimensional Au structures can be prepared on the designed scratching traces, providing a feasible path for fabricating high-quality metal-based sensors.

## Introduction

Metallic micro/nanostructures are widely used in sensors, catalysis, food security, high-precision circuit, energy, and other fields due to their excellent mechanical and physical performances [1–8]. For instance, the magnetic sensing technology based on metallic micro/nano coils exhibits significant advantages in the new generation of smart grid detection [9]. The increase in turns of metallic micro/nano coils in a limited volume allows them to exhibit a more uniform and dense magnetic field distribution during testing and working, thus achieving more excellent performance [10, 11]. On this basis, there is an urgent need for the preparation of high quality, low cost, and precise position metallic micro/nanostructures in order to achieve even better magnetic sensing performance in micro/nano magnetic sensors.

At present, the preparation methods of metallic micro/nanostructures mainly include sputter coating, electron beam deposition, evaporation, electroforming, electroless deposition, FIB cutting, nanowelding, atomic layer deposition, lithography, nanoimprinting, and ink direct writing [12–19]. However, traditional methods of obtaining metallic micro/nanostructures have many disadvantages such as difficult and unstable processes, complex preparation procedures, and high cost [20]. It is reported that copper (Cu) can be electrodeposited on nanosized grooves produced on silicon (Si) surfaces with a diamond-coated AFM tip at high forces [21]. The shape of metallic structures prepared by the method is relatively uniform and compact, and the process cost is low. Thus, it is a reliable method for preparing metallic micro/nanostructures. However, the applications are limited when using oxide masks due to strong chemical activity of solution composition. An alternative way is to pattern the self-assembled film-coated surface for metal deposition [16], while the chemically bonded film on Si surface makes it difficult to penetrate the mask by scratching [22]. In addition, the pre-scratching on Si substrates can easily induce the tip wear [23]. Therefore, a mask that can resist against the solution composition and be easily penetrated by the tip scratching is crucial for the solution-based preparation of metallic micro/nanostructures.

In this study, keto-aldehyde resin masks were used for directional preparation of metallic micro/nanostructures, which not only effectively reduced the tip wear during scratching, but also greatly simplified the preparation process of mask compared to surface oxide layers and self-assembled films. In addition, keto-aldehyde resins were easily soluble in organic solvents such as ethanol and acetone, but insoluble in water, which can effectively block metal deposition outside the scratch and can be easily removed after the metal structure formation. The mask ability of different film thickness was studied for the optimized deposition, and the influence of processing parameters (scratching load, number of cycles, and deposition time) on the formation of metallic micro/nanostructures was systematically investigated.

## Experimental

**Fig. 1:**
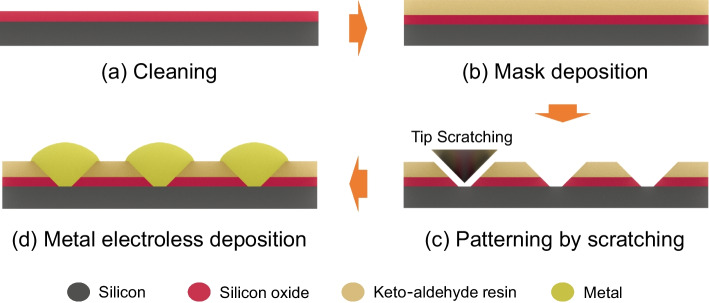
Schematic illustration showing the preparation process of metallic micro/nanostructures

The substrate used in this study is p-type Si(100) wafer (Suzhou Research Materials Microteach Co., Ltd.) with a thickness of ~ 0.5 mm and a root mean square roughness (RMS) of ~ 0.1 nm. Before the experiment, the Si(100) substrate was immersed in HF solution with a mass concentration of 20 wt% for 1 min to remove the natural oxide layer on the surface. After being washed with deionized water, it was transferred to Piranha washing solution (30 wt% hydrogen peroxide and concentrated sulfuric acid mixed solution with a volume ratio of 3:7) and heated for the reaction at 80 °C for 10 min to fully remove surface contaminations on Si substrate [24]. Then, the Si substrates were placed in acetone, anhydrous ethanol and deionized water in turn for ultrasonically cleaning for 10 min, respectively, and finally dried with N_2_ at room temperature for standby (Fig. 1a) [25].

The main process for the preparation of the keto-aldehyde resin mask is as follows. Firstly, the mixed solution of different concentrations of the resin and anhydrous ethanol was prepared with fully stirring. Then the Si substrate was placed on the polytetrafluoroethylene frame and slowly immersed in the solution for mask deposition. After the reaction time set in the experiment, the sample taken out by the tweezers was placed in vacuum drying oven to volatilize the excess solvent, thus completing the preparation of the mask (Fig. 1b). A home-made large-area scratching processing system was used to prepare scratches on the surface of the Si substrate deposited with the resin mask, and a constant normal load (10–50 mN) was applied to ensure that the mask in the scratched area was completely removed and the Si substrate was exposed (Fig. 1c). Subsequently, the patterned Si substrate was immersed in the metal salt solution (the mixed solution of 0.005 M chloroauric acid (HAuCl_4_) and 4.9 M hydrofluoric (HF) acid) for a period of time (60–150 s) to complete the directional deposition of the metal in-situ onto the scratches (Fig. 1d). Finally, the deposited substrate was immersed in anhydrous ethanol and deionized water to remove the keto-aldehyde resin to obtain a complete metallic structure.

Atomic force microscope (AFM; E-sweep, Hitachi, Japan) and metallographic microscope (Axio Lab.A1, Carl Zeiss, Germany) were used to characterize the topographies of metallic structures on Si surface. All AFM topographies were obtained by scanning in tapping mode using Si tip (NCHV, Veeco Instruments Inc, USA).

## Results and discussion

### Effect of resin concentration on mask thickness

**Fig. 2:**
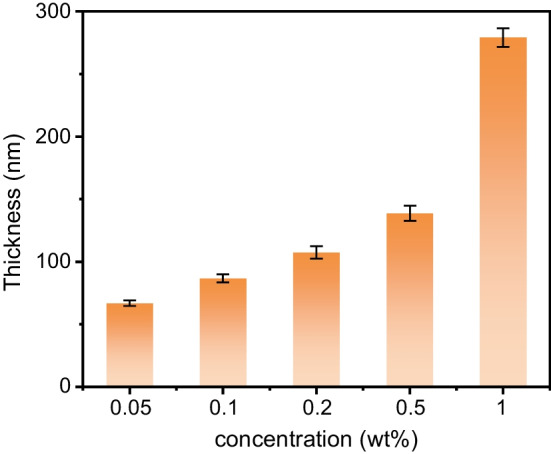
Comparison of keto-aldehyde resin mask thickness

The cleaned Si substrate was immersed in the keto-aldehyde resin solution with different concentrations for a period of time, and then taken out and dried in vacuum to complete the mask preparation. The mask thickness was measured using a spectral ellipsometer (M-2000V, J.A. Woolam, USA). Table 1 showed the mask thickness corresponding to different concentrations of the resin solution, and Fig. 2 demonstrated the comparison of mask thickness. It could be seen that the mask thickness was positively correlated with the concentration of the resin solution, and increased with solution concentration. Therefore, the mask thickness can be controlled according to the solution concentration.

**Table 1:** Mask thickness corresponding to different concentrations of keto-aldehyde resin solutions

Resin concentration solution (wt%)	Mask thickness (nm)
0.05	66.8
0.1	86.7
0.2	107.5
0.5	138.9
1	279.2

### Effect of mask thickness on tip scratching

**Fig. 3:**
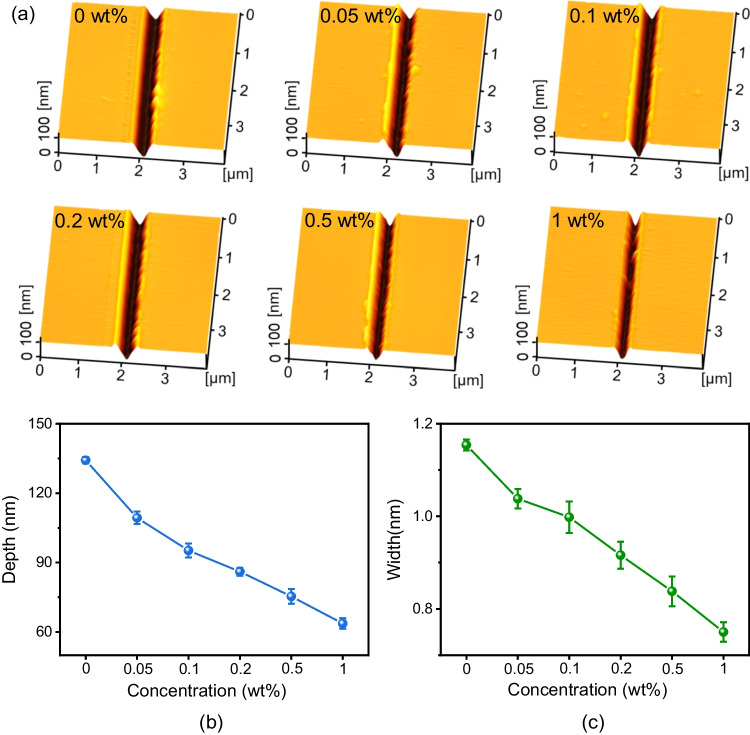
Scratches on keto-aldehyde resin mask-coated Si substrate. **a** Topographies of scratches on Si surface coated with different thickness of resin mask. **b** Comparison of scratches depth. **c** Comparison of scratches width

To expose the Si substrate by scratching, the tip should penetrate the mask under a given loading condition. The keto-aldehyde resin-coated Si substrates (Table 1) were firstly scratched with a diamond tip under 20 mN, respectively, and then immersed in anhydrous ethanol and deionized water to remove the resin mask. Since the hardness of the resin mask was much lower than that of the Si substrate, a normal load of 20 mN applied by the tip was sufficient to penetrate the mask and produce scratches on the Si substrate [26]. Figure 3 showed the AFM topographies of cleaned scratches on Si surfaces coated with different thickness of the resin. When the mask thickness increased from 66.8 to 279.2 nm, the depth of cleaned scratches gradually decreased from 134.22 to 68.62 nm, and the width decreased from 1.154 to 0.75 µm, as displayed in Fig. 3b and c.

### Selection of resin concentrations

The principle of metal deposition in solution on a semiconductor surface is as follows. Firstly, after immersing the scratched semiconductor substrate into a plating solution, the HF component in the solution will react with the oxide layer [27] in scratched areas immediately [16]. Due to the absence of external electrical contact during the ELD process, the etching of the oxide layer plays a key role in ensuring the sustainability of the metal deposition process on scratches [28]. Once the etching process is completed, a spontaneous redox reaction occurs between metal cations in solution and semiconductor atoms, i.e., the metal cations gain electrons and are reduced to metallic nuclei on the surface of scratches. The semiconductor atoms lose electrons and are oxidized then react with HF to form water-soluble fluorides to facilitate the continuation of the reaction [27]. Notably, Si substrate with attached metal nuclei has a higher electronic activity than that of Si atoms, which allows the reaction to proceed continuously, resulting in gradual growth of metal nuclei to form metal particles [26]. With increasing deposition duration, metal particles became interconnected to form metal structures. The above substrates were immersed directly into a metallic salt solution (a mixture of 0.005 M HAuCl_4_ and 4.9 M HF) in order to prepare the Au structures.

**Fig. 4:**
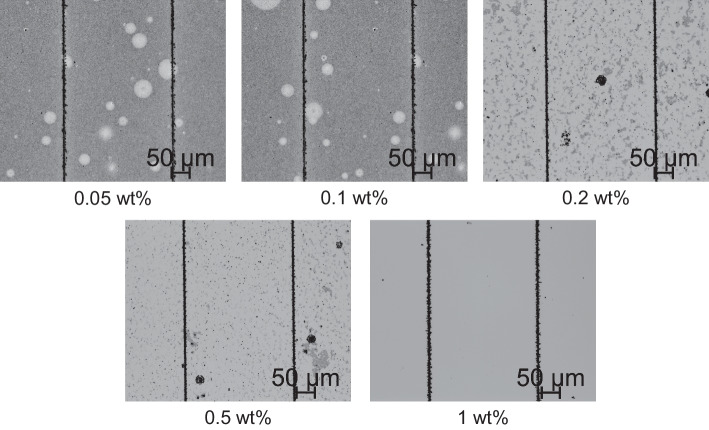
Comparison of the effects of keto-aldehyde resin masks with different thickness

As shown in Fig. 4, the mask prepared from 0.05 wt% and 0.1 wt% keto-aldehyde resin solution failed completely during the deposition process, resulting in metal deposition on the entire Si surface. Moreover, some obvious bubble holes were observed on the surface after the metal deposition. However, the mask prepared by 0.2 wt% and 0.5 wt% resin solution partially presented weak ability for assisting against the solution, leading to metal deposition in some areas besides the scratches. The mask prepared from 1 wt% resin solution worked well during the deposition process and the metal was only deposited in the scratched area. It can be deduced that the masks formed from the lower concentration of the keto-aldehyde resin solution were incompact, resulting in the reduction of metal cations to metallic simple substances by obtaining electrons in the gap. As the solution concentration increased, the mask gradually densified and the gaps were reduced. When the concentration of the resin solution used reached 1 wt%, there was no metal deposition outside the scratched area. Therefore, the1 wt% keto-aldehyde resin solution was used to prepare the mask in the following experiments.

### Effect of scratching parameters on metal deposition

**Fig. 5:**
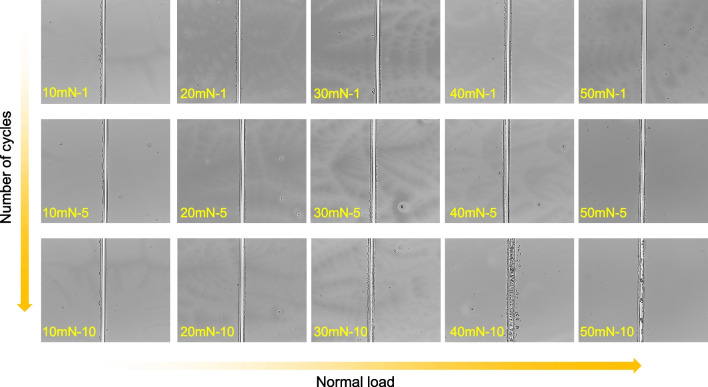
Scratch topographies on the surface of Si coated with keto-aldehyde resin mask under different normal loads and number of cycles

In order to prepare high-quality metal micro/nanostructures, the optimum scratching parameters were investigated. The effect of normal load and number of cycles in the scratching on the keto-aldehyde mask was revealed. Figure 5 illustrated the scratch topographies on the Si surface coated with the mask. These scratches were produced under different normal loads (10–50 mN) and scratching cycles (1–10) for comparison. The width of the substrate exposed by scratching gradually increased as the number of cycles increased under a given normal load. Meanwhile, with a certain number of cycles, the width of exposed Si substrate after the scratching would gradually increase with the normal load. It is worth noting that under the condition of multiple scratches with high normal load (40 mN and above), there will be a large amount of debris accumulation inside the scratch, which may affect the metal adsorption in redox reaction during the subsequent metal deposition process. Therefore, a load of less than 40 mN was used to explore the effects of the scratching parameters on metal deposition during the subsequent experiment.

**Fig. 6:**
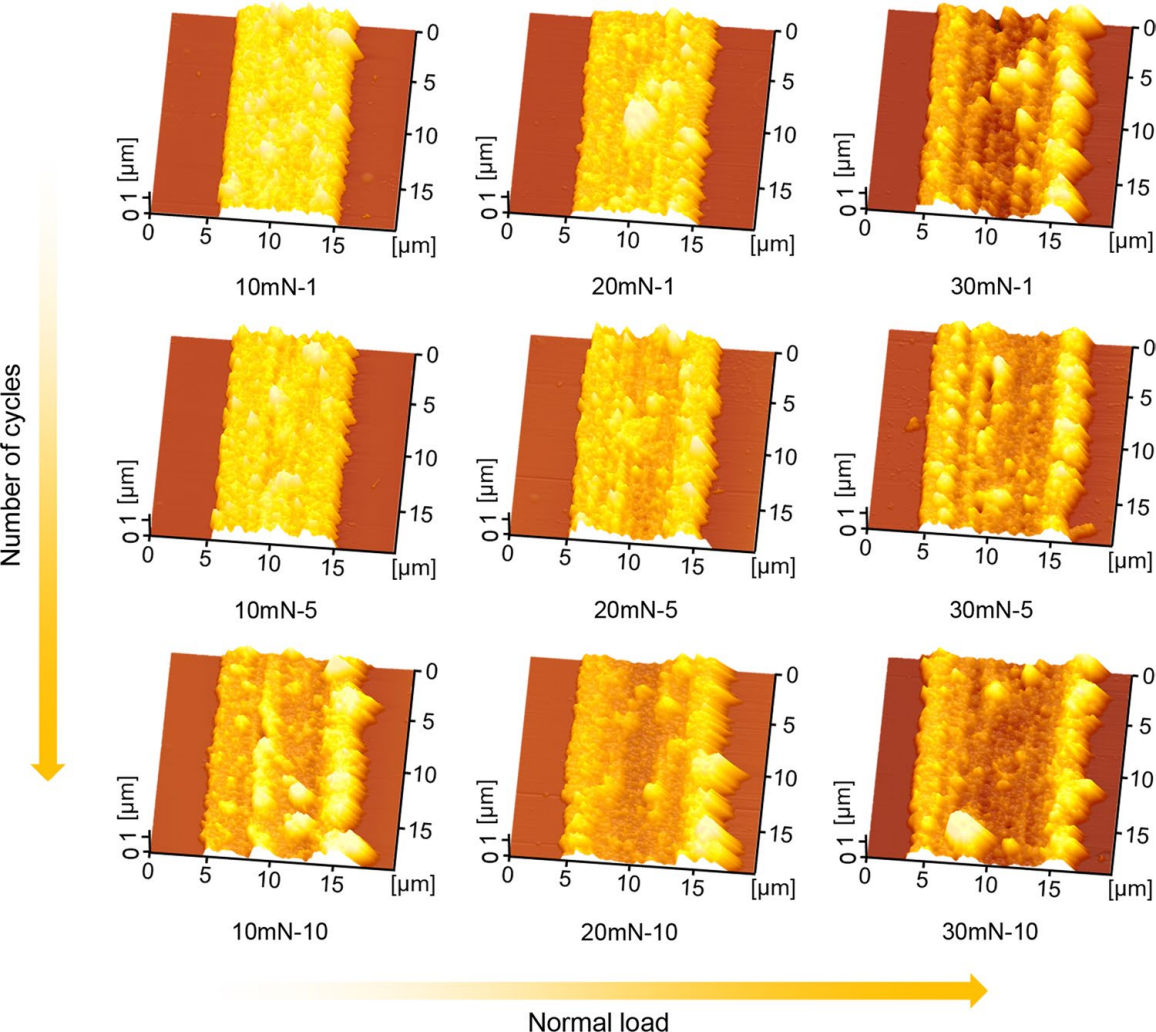
Effect of scratching parameters on metal directional deposition

The substrates with different scratches were immersed directly in the mixed solution of 0.005 M HAuCl_4_ and 4.9 M HF for 120 s for Au deposition onto the scratched area, and then taken out for further detection with the AFM. Figure 6 showed the comparison of Au line-structures formed on different scratches. It can be seen that with the increase of normal load and number of cycles, the width and surface undulation of Au line-structures increased gradually. Large particles appeared on the scratches produced under high loads and multiple cycles, which may be closely related to the crystalline growth process. According to previous reports, the deformation (crystal defects) can be detected on Si subsurface during scratching due to frictional shear from the diamond tip under high loads [27]. Notably, the defects will interfere with electron migration on the scratch, thus affecting the redox reaction [29]. As a result, one possible mechanism is that the distribution of crystal defects in the scratched area is not homogeneous (especially scratches induced by high loads and multiple scratching), which leads to a difference in the rate of electron migration and thereby changes the topography of Au line-structures. Moreover, the particles on the surface of Au line-structures are randomly distributed and have not grown in a specific direction. Thus, it can be deduced that Au structures deposited on Si surface nanoscratches are composed of polycrystalline Au [30]. In summary, provided that the mask can be scratched through to expose the underlying Si surface, a single-pass scratch with a lower normal load can facilitate the deposition of metals on the scratch and obtain a higher quality metallic structure.

### Effect of deposition time on metallic structure preparation

**Fig. 7:**
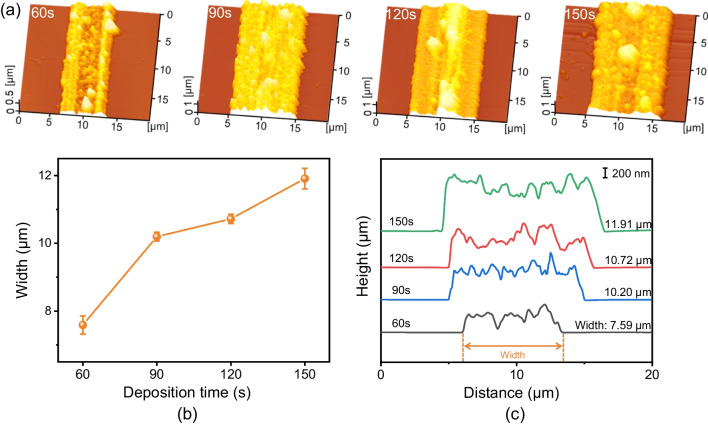
Deposition time-dependent Au structures on Si substrate. **a** AFM topographies of Au structures at different deposition times. **b** Comparison of the width of Au structures. **c** Comparison of cross-sectional profiles of Au structures

**Fig. 8:**
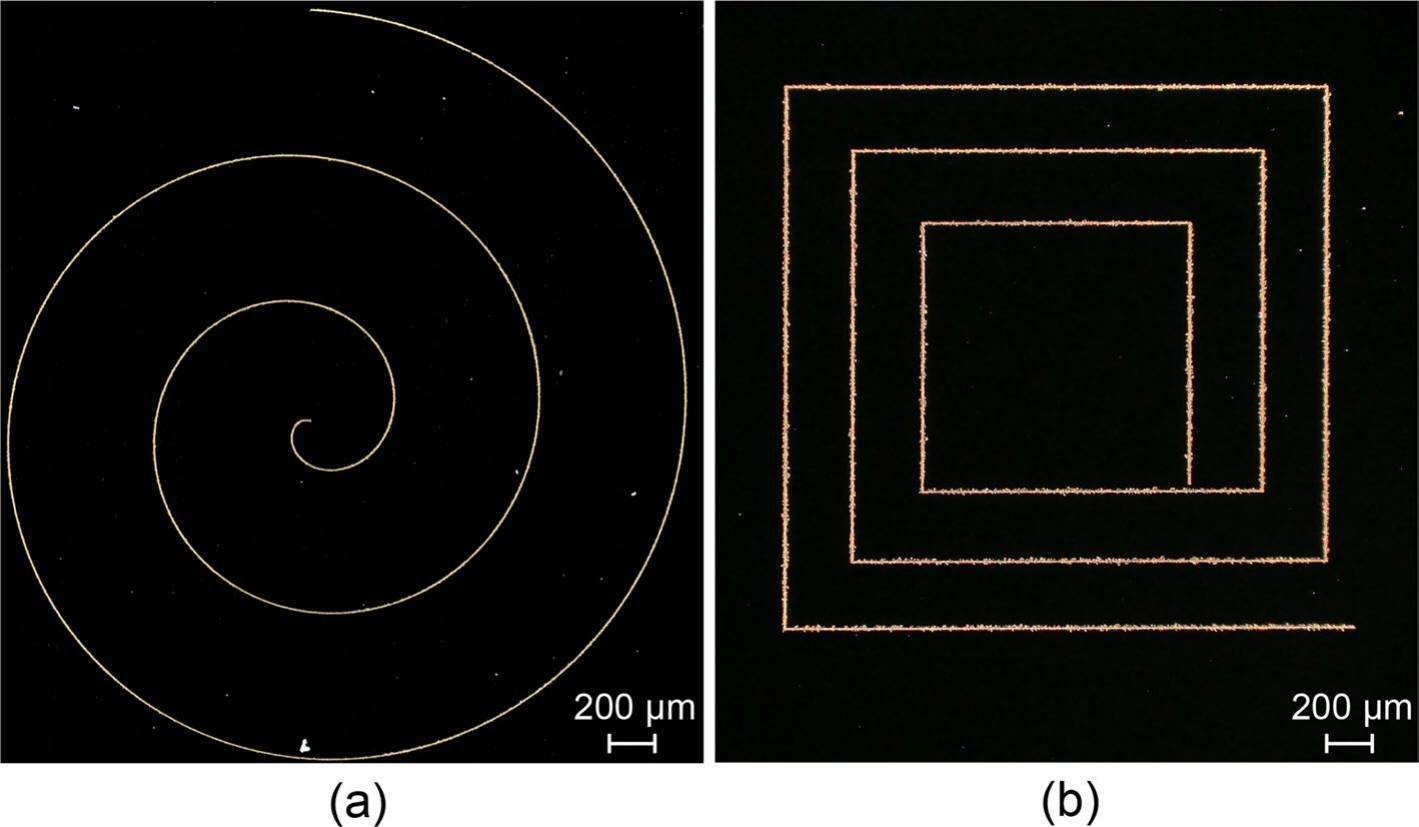
A spiral coil (**a**) and a rectangle (**b**) of Au structures prepared by scratch-induced deposition on Si substrate

Figure 7 showed AFM topographies of Au structures deposited under different times, which were obtained by soaking in a mixed solution of 0.005 M HAuCl_4_ and 4.9 M HF. As shown in Fig. 7b, the width of the metallic structure gradually increased with deposition time. Combining Fig. 7a and c, it could be obtained that with the same mask thickness, normal load and number of cycles, the metallic structure was more uniform when immersed in the mixed solution for 90 s. Based on above experimental results, the optimized experimental parameters were adopted to fabricate large-area complex textures on Si surface. Figure 8a showed the surface topographies of prepared planar spiral metal coils, where a diamond tip with a cone angle of 90° was employed to remove the mask. It can be seen that the metal structure prepared by this method presented a good continuity, and the width is ~ 10 μm. Notably, Au rectangle pattern fabricated by scratch-induced deposition structure exhibited a larger width under the same condition, where the scratch was obtained by a diamond tip with a cone angle of 120° (Fig. 8b). In fact, the controllable preparation of metal structures is expected to be achieved through the rational programming of the trajectory scratching path under a given condition.

## Conclusions

In this paper, the effects of keto-aldehyde resin masks and scratching parameters on the deposition behavior of Au on Si surface were investigated based on scratch-induced metallic micro/nanostructure formation in the mixed solution. The main conclusions obtained are as follows.

1. In the process of metal deposition, keto-aldehyde resin mask with a certain thickness can effectively protect the unscratched area of the substrate and facilitate metal deposition in-situ on the scratch.
2. Once the keto-aldehyde resin mask is completely scratched through and the underlying Si substrate is exposed, a single-pass scratch with a lower normal load is more conducive to induce the formation of metallic structures on the scratched area.
3. By optimizing the scratching and deposition parameters, the preparation of micro/nanostructures with arbitrary two-dimensional patterns can be achieved.

## Data Availability

All the data are available from the corresponding author on reasonable request.
